# Implications of rituximab pharmacokinetic and pharmacodynamic alterations in various immune-mediated glomerulopathies and potential anti-CD20 therapy alternatives

**DOI:** 10.3389/fimmu.2022.1024068

**Published:** 2022-11-07

**Authors:** Jan Miroslav Hartinger, Vojtech Kratky, Zdenka Hruskova, Ondrej Slanar, Vladimir Tesar

**Affiliations:** ^1^ Department of Pharmacology, First Faculty of Medicine, Charles University and General University Hospital Prague, Prague, Czechia; ^2^ Department of Nephrology, First Faculty of Medicine, Charles University and General University Hospital Prague, Prague, Czechia

**Keywords:** ANCA-associated vasculitis, membranous nephropathy, lupus nephritis, minimal change disease, ocrelizumab, vedolizumab, ofatumumab, obinutuzumab

## Abstract

The specific B-cell depleting anti-CD20 monoclonal antibody rituximab (RTX) is effective in terms of the treatment of various immune-mediated glomerulopathies. The administration of RTX has been shown to be reliable and highly effective particularly in patients with ANCA-associated vasculitis, which is manifested predominantly with non-nephrotic proteinuria. Stable long-term B-cell depletion is usually readily attained in such patients using standard dosing regimens. However, in patients with nephrotic syndrome and non-selective proteinuria, the RTX pharmacokinetics is altered profoundly and RTX does not maintain high enough levels for a sufficiently long period, which may render RTX treatment ineffective. Since complement-derived cytotoxicity is one of the important modes of action of RTX, hypocomplementemia, frequently associated with systemic lupus erythematodes, may act to hamper the efficacy of RTX in the treatment of patients with lupus nephritis. This review provides a description of RTX pharmacokinetics and pharmacodynamics in several selected glomerulopathies, as well as the impact of proteinuria, anti-drug antibodies and other clinical variables on the clearance and volume of distribution of RTX. The impact of plasmapheresis and peritoneal dialysis on the clearance of RTX is also discussed in the paper. A review is provided of the potential association between pharmacokinetic and pharmacodynamic alterations in various kidney-affecting glomerular diseases, the sustainability of B-cell depletion and the clinical efficacy of RTX, with proposals for potential dosing implications. The role of therapeutic drug monitoring in treatment tailoring is also discussed, and various previously tested RTX dosing schedules are compared in terms of their clinical and laboratory treatment responses. Since alternative anti-CD20 molecules may prove effective in RTX unresponsive patients, their pharmacokinetics, pharmacodynamics and current role in the treatment of glomerulopathies are also mentioned.

## Introduction

The first anti-CD20 chimeric mouse/human monoclonal antibody (MAB) rituximab (RTX) was originally developed in the 1980s and 1990s for the treatment of hematologic malignancies. The Food and Drug Administration (FDA) first approved the use of rituximab to treat B-cell non-Hodgkin’s lymphoma in 1997, following which rituximab obtained FDA approval for the treatment of chronic lymphocytic leukemia, rheumatoid arthritis (RA), granulomatosis with polyangiitis (GPA), microscopic polyangiitis (MPA) and pemphigus vulgaris. In addition, numerous studies have found rituximab to be effective for other autoimmune diseases including systemic lupus erythematosus (SLE), primary membranous nephropathy (MN) and immune thrombocytopenia (1–3).

**Figure 1:**
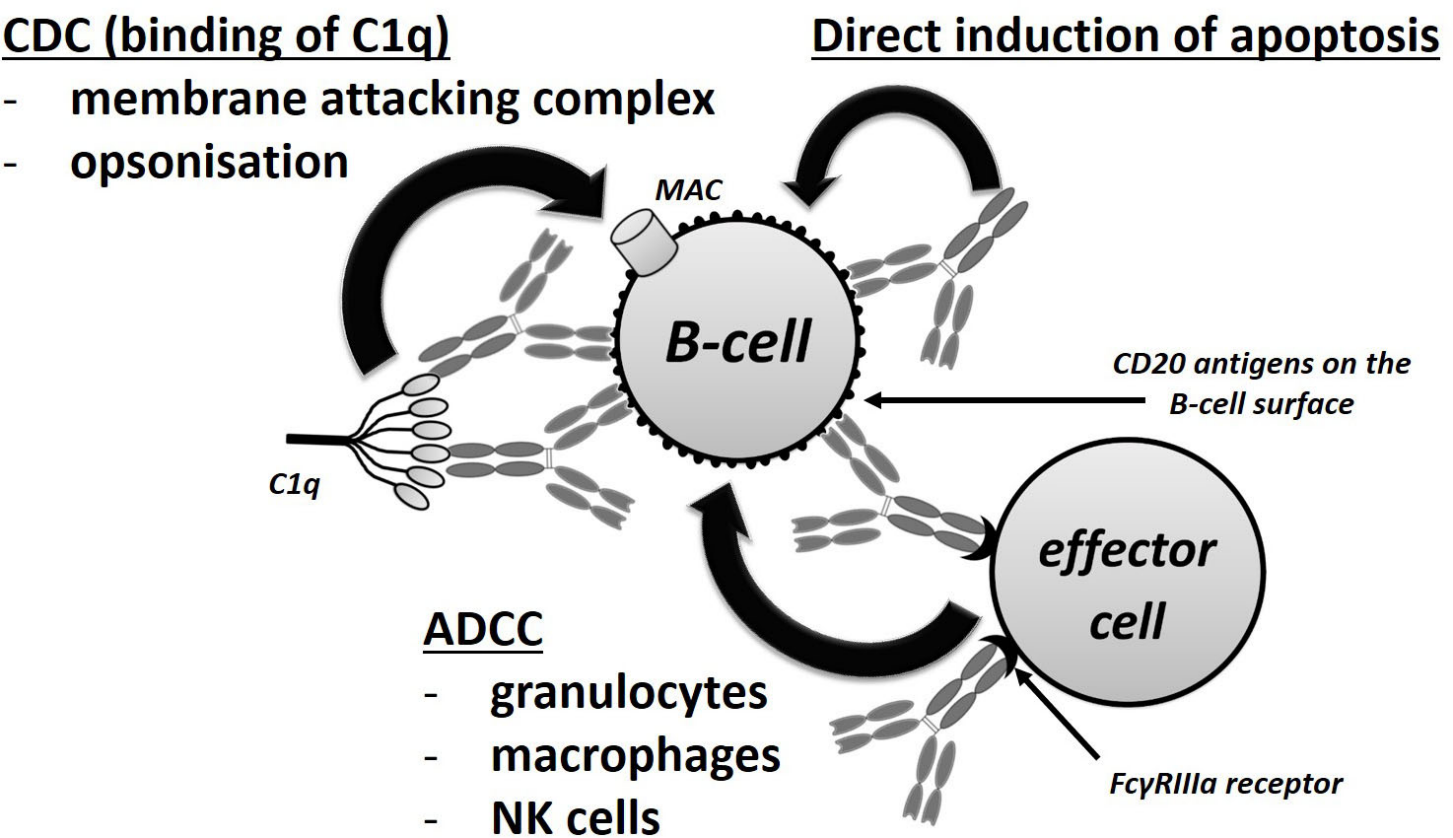
Mechanisms of action of rituximab. CDC, complement-dependent cytotoxicity; ADCC, antibody-dependent cellular cytotoxicity.

Rituximab is a chimeric MAB that is targeted at the CD20 transmembrane protein, which is present on all B-lineage cells other than precursor B cells and differentiated plasma cells (4). By binding to the CD20 protein, rituximab triggers cell-mediated and complement-mediated cytotoxicity that results in the depletion of CD20 positive B cells (5, 6). A simplified scheme of the anti-CD20 antibody mechanism of action is presented in **Figure 1**. The absence of the expression of CD20 in pre-B hematopoietic stem cells conserves the stem cell pool, which is important in terms of B cell reconstitution following the depletion thereof. The production of antibodies by differentiated plasma cells is also not influenced by the administration of rituximab (7) and, therefore, B-cell depletion does not *per se* affect the antibody levels in RTX-treated patients (8). It was also suggested that RTX binds to sphingomyelin-phosphodiesterase-acid-like-3b protein on the surface of podocytes and prevent their apoptosis, but relevance of this mode of action has been subsequently questioned as other anti-CD 20 molecules that do not exert this mode of action was also proved to be effective in treatment of glomerulopathies (9).

It has been proven that RTX pharmacokinetics is influenced by several clinical covariates, namely the severity of the disease, antigen derived factors (e.g. the amount of antigen in the tissues), gender, body weight and the selectivity of proteinuria (PU), which may act to alter the response to treatment (10). Moreover, the potential formation of anti-RTX antibodies may result in decreased treatment effectivity (11, 12). Therefore, the issue of immunogenicity is one of the reasons for the development of alternative anti-CD20 MABs in recent years (13). This review focuses on RTX and other anti-CD 20 antibodies used in the treatment of immune-mediated nephropathies, particularly on the characteristics of the pharmacokinetics and their potential influence on the effectiveness and safety of RTX therapy.

## Overview of anti-CD20 monoclonal antibodies

A second generation of humanized and fully human anti-CD20 MABs with improved CD20-depleting properties was developed following the success of rituximab (**Table 1**), including ocrelizumab, veltuzumab and ofatumumab. All of these antibodies are IgG1 anti-CD20 type I (rituximab-like) molecules with intentionally modified pharmacodynamic profiles (14). Ocrelizumab binds to a different but overlapping CD20 epitope than rituximab, and the Fc fragment of ocrelizumab exhibits increased binding affinity for low-affinity FcγRIIIa receptor variants on effector cells. Since low-affinity FcγRIIIa receptor variants are associated with the lower effectivity of RTX treatment, ocrelizumab potentially represents a treatment alternative for certain RTX resistant diseases (15). Moreover, ocrelizumab induces a lower degree of complement-dependent cytotoxicity (CDC) than does rituximab (14, 16, 17). Veltuzumab has a similar binding epitope as rituximab (18), differing from RTX with concern to just one amino-acid in the variable region, which results in a higher level of CD20-binding avidity and a stronger complement activating effect in certain cell lines compared to rituximab (14). Ofatumumab is a fully human antibody that binds to a different epitope than rituximab and other anti-CD 20 antibodies (19). It effectively lyses the cells in RTX resistant cell lines and is more effective than rituximab in terms of the induction of CDC due to the binding site being located closer to the cell membrane (6, 14). It also evinces a longer residence time than RTX, i.e. it dissociates more slowly once it has bound to CD20 (20). This is crucial with respect to C1q binding and CDC activation (21).

**Table 1:** Characteristics of anti-CD20 monoclonal antibodies.

Generation	Antibody	Antibody type	Format	Comparison to rituximab
1^st^	Rituximab	I	Chimeric IgG1	
2^nd^	Ocrelizumab	I	Humanized IgG1	CD20 epitope overlapping with RTX, increased binding to FcγRIIIa and decreased CDC
	Veltuzumab	I	Humanized IgG1	Similar CD20 binding epitope as RTX, greater binding activity and increased effect on CDC
	Ofatumumab	I	Human IgG1	Binding to different CD20 epitope than other anti-CD20 molecules, strong CDC, slower dissociation from CD20 than RTX
3^rd^	Obinutuzumab	II	Humanized IgG1	CD20 epitope overlapping with RTX, do not aggregate CD20 on cell surface, binds to B-cells in lower density, strong ADCC and direct cell-killing, diminished CDC

ADCC, antibody-dependent cellular cytotoxicity; CDC, complement-dependent cytotoxicity; RTX, rituximab.

The third generation of MABs includes obinutuzumab, a humanized de-fucosylated IgG1 monoclonal type II antibody against CD20, which differs from type I molecules *via* its differing ability to redistribute CD20 molecules on the cell surface and its differing pattern of Fc fragment mediated effector mechanisms (18, 22). Because the CD20 epitope of obinutuzumab overlaps with that of RTX, it allows it to bind to the B-cell surface only at a lower density than type I molecules (18). The glycoengineered Fc fragment enhances its binding affinity to the FcγRIII receptor, thus resulting in the more potent induction of direct cell death (DCD) and more effective antibody-dependent cellular cytotoxicity (ADCC) than rituximab. Conversely, obinutuzumab induces a markedly reduced CDC (up to 100-fold compared to type 1 antibodies) and does not localize the antibody-antigen complex into lipid rafts (6, 23). More detailed information on the characteristics of newer generations of anti-CD20 MABs that are beyond the scope of this review is provided elsewhere (6, 13, 14, 18).

## Rituximab as a therapy for immune-mediated glomerulopathies

According to KDIGO 2021 guidelines, RTX is indicated for the treatment of AAV, MN, relapsing minimal change disease (MCD) and certain cases of lupus nephritis (LN). The suggested dosing schedules consist of either body-surface area (BSA) based (375 mg/m^2^ weekly for 1-4 weeks) or fixed dosing (two 1000 mg doses administered two weeks apart) (24–26). The recommendation of these two differing schemes mirrors a certain level of uncertainty regarding the dosing of RTX since the two regimens have been proposed arbitrarily and could lead to significant differences in the overall exposure to RTX in some patients.

Compared to previous treatment modalities (i.e. cyclophosphamide and corticosteroids followed by maintenance therapy with azathioprine), RTX has been shown to be similarly effective in terms of inducing AAV remission, and superior in terms of maintenance treatment (27–29). Moreover, RTX-treated patients with AAV do not exhibit an enhanced malignancy risk in contrast to cyclophosphamide-treated patients and require a lower number of infusions, which acts to decrease the overall treatment burden (30). Therefore, RTX has been declared a potential first line treatment modality for AAV according to KDIGO guidelines (24) and has been officially approved with respect to this indication as an induction therapy in the form of four weekly-administered 375 mg/m^2^ doses. More recent studies have proven that RTX is also effective in AAV maintenance therapy (31). The meta-analysis of RTX treatment in MN evinced at least the same efficacy as the control group (pooled data from studies applying cyclosporine, alkylating agents and other treatments) with respect to survival, total remission, PU reduction, increases in the serum albumin level and renal function decline, and evinced improved efficacy in terms of inducing complete remission (32). Current KDIGO guidelines indicate that RTX should be considered as a first line treatment for MN patients with a moderate to high risk of kidney function deterioration (24, 33). The randomized controlled trial (RCT) of RTX for frequently relapsing or steroid dependent NS in children was terminated prematurely due to interim analysis that indicated significant improvement in the active arm, which rendered the randomization to a placebo arm no longer ethical (34). Moreover, RTX has been proven to be an effective treatment modality for adults with MCD in terms of reducing the number of relapses and PU reduction (35, 36), and is currently recommended by KDIGO for relapsing MCD (24). Much less data is available for RTX effectivity in focal segmental glomerulosclerosis (FSGS) (25). Whereas MCD and FSGS have been initially considered a T-cell mediated diseases (37), the therapeutic efficacy of anti-CD20 antibodies has highlighted a pathogenic role also for B cells, and several studies on the role of total and specific B cell subsets have been described in the last 15 years, especially in children, as recently reviewed by Colucci et al. (38). In particular, the association of the response to RTX with the recovery of specific B cell subsets more than of total B cells has been proved in several studies (39–41). Furthermore, anti-CD20 treatment could directly or indirectly (via B-cell depletion) restore T-cell homeostasis in patients with frequently relapsing and steroid dependent nephrotic syndrome (40, 42, 43). With respect to LN, RTX is regarded as a potential modality when 1^st^ line treatment fails to bring the disease under control (24). The data for RTX in the treatment of LN are inconclusive. There is only one RCT of RTX vs placebo available. In this study RTX on top of mycophenolate mophetil and corticosteroids did not improve the treatment efficacy (26, 44). This rendered firm recommendations impossible, even though some authors consider RTX to be a relatively effective treatment modality based on the results of non-randomized trials (45). Nevertheless, in contrast to MCD, the response in LN appears to correlate with the effective depletion of B-cells (46, 47). Finally, RTX has been proven to be ineffective in IgA nephropathy in an RCT, despite the effective depletion of B-cells over 12 months (48).

## Safety of RTX

According to over two decades of clinical experience, RTX has been proven to be a safe treatment modality, with the most frequent adverse reactions being related to the infusion procedure; such reactions have been observed in up to a quarter of patients, especially during the first infusion, and are usually mild to moderate in severity (e.g. headache, skin itchiness, throat irritation). The release of cytokines plays a pivotal role in the development of such reactions (49, 50).

The application of RTX increases the risk of *de novo* infections, which most commonly affect the ears, nose and upper respiratory tract; more rarely the administration of RTX is complicated by the development of urinary infection, septicemia, prostatitis, colitis, pyelonephritis and other infections (51). The reactivation of chronic hepatitis B and C infection may occur following exposure to RTX and, therefore, prophylaxis should be initiated for those patients with a high risk of reactivation. Severe infections such as pneumocystis pneumonia and progressive multifocal leucoencephalitis are extremely rare (25). Nevertheless, Li et al. described in their network meta-analysis that RTX may cause more infections than tacrolimus and other immunosuppressants during the treatment of LN based on one retrospective cohort trial and one non-randomized controlled trial (45). In the case of AAV, pneumocystis prophylaxis with low-dose trimethoprim-sulfamethoxazole is advised for 6 months following rituximab induction due to the lungs affecting the nature of the disease (24). RTX therapy also leads to a diminished humoral response to vaccination, including to the SARS-CoV-2 vaccine (52). Thus, if possible, vaccination should be performed prior to the administration of RTX.

A further safety concern relates to hypogammaglobulinemia, which has been observed to develop in a significant proportion of patients receiving RTX. Low baseline serum IgG levels represent the main risk factor for the development of hypogammaglobulinemia during RTX therapy. The monitoring of immunoglobulin levels is recommended and, in the case of recurrent infections, immunoglobulin replacement therapy should be provided (8, 53). Late onset neutropenia (4 or more weeks following the administration of RTX) is a further relatively common and often incidental adverse reaction; however, since it may be associated with serious infections, it requires careful monitoring (54). Risk factors for development of late onset neutropenia are older age, more advanced disease and co-medication with antimetabolites in patients with B-cell lymphoma. Interestingly the incidence of late onset neutropenia in these patients did not correlate with number of treatment courses and therefore with the overall cumulative RTX dose (55). It can therefore be expected, that also various dosing schedules used during treatment of autoimmune diseases possess similar risk of late onset neutropenia as this adverse effect is most probably not dose-dependent.

Other observed adverse reactions include gastrointestinal and hepatic disorders, cardiovascular disorders, hematological events, musculoskeletal disorders and nervous system disorders (51). Progressive multifocal leukoencephalopathy comprises one of the most serious adverse effects of RTX (as well as other immunosuppressants); fortunately, however, it occurs only extremely rarely (56).

## The pharmacokinetics of rituximab and the covariates that influence it

The pharmacokinetics of RTX in patients differs according to disease type and activity (57), with one of the most altered pharmacokinetic profiles having been described in MN patients during periods with high levels of non-selective proteinuria (11). Pharmacokinetic parameters described in different studies vary due both to the different diseases studied and the different pharmacokinetic models and analytical methods applied (10). Therefore, Bensalem et al. used concentration-time data from 5 different studies and applied the same method to calculate RTX pharmacokinetic characteristics. Than they compared pharmacokinetic characteristics of five different diseases and found out that there are profound differences between autoimmune and hematological diseases (57). Generally, RTX (as well as other MABs) pharmacokinetic is best described by means of a two compartmental model with a central plasmatic compartment and a peripheral tissue compartment with the occurrence of elimination in the central or both of these compartments (10, 11, 58, 59). During the treatment of most autoimmune diseases RTX has relatively stable half-life whereas its half-life in hematologic diseases typically prolongs during the treatment as the excessive number of target cells decrease (60).

### Absorption

RTX is administered i. v. or s. c. with comparable degrees of efficacy, with an increased number of non-severe administration-related reactions being associated with the s. c. administration route. Since a higher number of infusion-related reactions are associated with the first administration of RTX, it is considered prudent to administer the first dose *via* the intravenous route so that it can be discontinued immediately should it be necessary (61).

Following s. c. administration, absorption occurs mainly *via* convective flow into the lymphatic vessels. In animal studies, the s. c. bioavailability (BAV) was found to be 1.9 times higher following the addition of hyaluronidase; therefore, recombinant human hyaluronidase is added to the s. c. preparation of RTX so as to enhance the absorption process. Some patients develop antibodies against this enzyme, which is of unknown clinical relevance (6, 61). According to clinical studies, the s. c. BAV of RTX is 50-100%, and the peak plasmatic concentration occurs 2-8 days following administration (10, 58).

A fixed s. c. dose of 1600 mg was shown to evince comparable exposure to a BSA calculated i. v. dose of 500 mg/m^2^ in a study with CLL patients. The 5^th^ percentile of exposure was almost the same for both dosing strategies, whereas the 95^th^ percentile was higher for s. c. administration. This proves that patients up to a BSA of 2.4 m^2^ (the largest patient in the 1600 mg group) would not be underdosed with an s. c. 1600 mg fixed RTX dose (6). Nevertheless, concerning fixed dosing, smaller patients have a higher exposure and it has not been proven that patients with a BSA of over 2.4 m^2^ would not be underdosed.

### Distribution

The volume of distribution of therapeutic antibodies is usually relatively low, with a limited distribution to the extracellular compartment (10). RTX, as well as other antibodies, does not cross the blood brain barrier and must be administered intrathecally in the treatment of CNS lymphomas. RTX binds to the CD20 B-lymphocytes in plasma, the lymph nodes and bone marrow. After reaching the tissue capillaries, RTX is distributed slowly to the tissues by means of transcytosis and paracellular convective pathways. The RTX volume of distribution is 9.6 L of which approximately one third is plasma and the rest is the tissue compartment (58). Differing volume of distribution values have been published according to the simplification of the pharmacokinetic model (even with very low volumes of distribution and clearance in non-compartmental analysis) (62). The RTX volume of distribution is significantly larger in males with AAV, RA and lymphomas (63, 64). With concern to children, both the volume of distribution and clearance were found to correlate positively with the BSA according to one study on patients with frequently relapsing and steroid dependent NS (65). It is well known that a higher tumor burden in lymphomas represents a higher number of CD20 molecules that bind more RTX in the tissues and, finally, this could be interpreted as an increase in the apparent volume of distribution or clearance of free RTX (66, 67). Nevertheless, it would be more appropriate to interpret it as target-mediated clearance, which decreases following the depletion of B-cells (58, 63, 68).

RTX passes into ascitic fluid (or the peritoneal dialysate). 0.8 mg/L of RTX was detected in ascites 3 hours following i. v. administration and 3.3 mg/L was detected after 24 hours in patients with lymphoma and repeatedly drained ascitic fluid, with the disappearance of CD20 positive cells from the ascitic fluid (69). A case report has described the distribution of RTX into the pleural fluid (70).

RTX, as well as other molecules equipped with the Fc fragment, crosses the placenta and is concentrated in the fetus during the third trimester. In three cases, RTX levels and B-cell counts were measured in newborns following RTX administration during the third trimester. In two cases, the newborn RTX levels were higher than the mother’s, and in all cases their B-cells were depleted at the moment of delivery with subsequent full repopulation without any clinical consequences (71).

### Elimination

The terminal elimination half-life of RTX is approximately 3 weeks in hemato-oncologic and autoimmune diseases that do not affect the kidneys. A study by Regazzi et al. reported that RTX levels 6 weeks following the last infusion were not statistically significantly different in group of patients with various autoimmune diseases and patients with follicular lymphoma with a low tumor burden (66).

The primary constitutive RTX elimination route consists of the uptake by cells of reticuloendothelial system (RES). Following absorption from plasma *via* constitutive pinocytosis, plasmatic proteins are degraded in the lysosome, provided that the FcRn (Brambell) receptors do not preserve them. MABs bind the FcRn receptor *via* their Fc fragment and, subsequently, they are released from the cell instead of being degraded, which acts to markedly prolong their half-life, i.e. to around 21 days (58). A further RTX clearance pathway comprises its binding to its target antigen (target-mediated elimination) especially at the beginning of the treatment of hematologic diseases with a high tumor burden. Target-mediated clearance is a non-linear process and slows down with the depletion of the B-cells. Therefore, if the patient has a high number of B-cells, the first dose of RTX rapidly binds to CD20 and is eliminated more rapidly from the plasma than after subsequent doses (58, 63).

It is worthy of note that CD20 (in contrast to CD19 and other surface molecules) do not rapidly internalize after they have been bound by antibodies; therefore, target-mediated degradation inside the targeted cells most likely plays a minor role in RTX. Nevertheless, shortly after RTX is infused, monocytes/macrophages may begin to “pick up” the antibody binding CD-20 antigen without destroying the entire B-cell. This so called “shaving” or trogocytosis process may act to rapidly lower the number of CD20 molecules on the B-cells as soon as after 1 hour following the infusion of RTX (58, 72).

## Factors that influence the clearance of RTX

### Number of target antigens

During NHL and CLL treatment, RTX (as well as obinutuzumab) has been shown to have a clearance that is combined of time-independent and exponentially decreasing processes, which mirrors its constitutive degradation and rapid binding to the CD20 antigen and the subsequent depletion of the B-cells (73). Clearance is, therefore, modified in terms of its exponentially decreasing component by the number of target molecules (the antigen sink), and the tumor burden plays an important role in the clearance of RTX during the treatment of hematological diseases. Low RTX levels allow for the prediction of hemato-oncologic disease relapses, which may suggest that the affected patients were underdosed for their particular tumor burden (58). RTX clearance is also enhanced in the case of a higher B-cell count in RA, and non-linear pharmacokinetics have also been proven in AAV, which is described in more detail in the text below (74).

### The overall IgG level

The higher the IgG levels in the blood of the patient, the more FcRn receptors are settled, which renders the half-lives of the antibodies shorter. Moreover, since the ADCC depends on low-intermediate affinity to FcγR on the effector cells, an excess of other IgGs may reduce the ability of RTX to induce ADCC (58).

### Gender

At least with concern to DLBCL treatment, women have a lower RTX clearance, with a half-life of 30.7 days compared to 24.7 days in males. This indicates a longer exposure time to RTX for women, accompanied by a consequently better treatment response (58, 64).

### BSA

With respect to children, clearance correlated positively with BSA in one study with frequently relapsing or steroid dependent NS (65).

### Anti-drug antibody (ADA) formation

Since MABs are large exogenous molecules, they are capable of inducing the production of antibodies against themselves. Combination of murine containing variable domain amino-acid sequences with human constant domains led to reduction of immunogenicity in chimeric antibodies and this should be more pronounced in humanized or fully human antibodies. Nevertheless, many other factors influence the MAB immunogenicity, e. g. impurities in the medicinal product, glycosylation with non-human glycans and the disease-type (75). If ADAs are targeted against the variable domain of RTX, they may act to completely neutralize its effect. Provided the ADAs do not compromise the RTX binding capacity for CD20, they may influence only the RTX elimination rate since immune complexes can be cleared more rapidly than free MABs. The frequency of the formation of ADA described in several clinical studies with MABs is between 0-66% (11, 12, 34, 75). This wide variation is not only the result of the differing immunogenicity of the administered MABs, but is largely due to differences in the methodology applied for the determination of the ADA. According to a limited study involving 17 SLE patients (7 with LN) treated with RTX, ADA were detected in 11 of them, namely in those patients with a higher baseline disease activity and African Americans. A more rapid decrease in RTX levels was observed in patients with high ADA titers (76). One of the technical challenges in terms of the determination of ADA is that it may become detectable for the first time only following the complete elimination of the RTX since the laboratory method is only able to detect unbound ADA. Thus, Boyer-Suavet et al. detected unusually high levels of anti-RTX ADA (23%) in patients with MN compared to other studies when they measured ADA levels 6 months following the last administration of RTX. Interestingly, the ADA levels were found to be unmeasurable 3 months following the administration of RTX in all the patients in their study (12).

### Proteinuria

The clearance of RTX is also enhanced *via* non-selective proteinuria, as suggested by a study in which a positive correlation between the clearance and the degree of PU was observed in patients with MN (11). Jacobs et al. detected rituximab in the urine of 12 patients with nephrotic diseases (77), and the effect of PU on the elimination of RTX was also directly proven in two case reports in which the authors measured the urinary RTX concentrations in patients with nonselective PU. The highest concentration of RTX in the urine was approximately 3.5 mg/L, and the RTX/creatinine ratio correlated with the selectivity of the PU. The same study reported that no RTX was detected in the urine of three patients with glomerular diseases that were not associated with proteinuria (78). According to one pediatric case report concerning steroid resistant NS with extremely high non-selective PU, the RTX half-life was extremely short, i.e. less than one day, with unmeasurable levels 4 days following the initial 375 mg/m^2^ dose. The authors measured RTX levels in the urine of the patient and calculated that the urinary clearance was as high as 25% of the total body clearance. Nevertheless, an increased RTX loss into the urine does not sufficiently explain all the differences evident in the RTX pharmacokinetics of nephrotic patients (70).

### Hemodialysis and therapeutic plasma exchange.

Hemodialysis exerts no effect on the elimination of RTX; indeed, RTX levels have been observed to be slightly elevated following an HD session due to the hemoconcentration (79). Therapeutic plasma exchange (TPE) is an effective RTX (and other antibodies) elimination route (59, 80). The earlier the TPE is performed following the infusion of RTX, the higher the eliminated amount of RTX. When TPE is performed 24-72 hours following the i. v. infusion of RTX, 47-54% of the administered dose is removed, with the removal of only 9-26% *via* TPE 6 days following RTX administration (59). This is in line with the expectation that more RTX is removed prior to the completion of the distribution phase. This most probably also renders s. c. administration less prone to the influence of TPE even though no formal studies have been performed to prove this assumption.

### Peritoneal dialysis

Peritoneal dialysis was described as an effective RTX elimination route in one case report in which a patient treated with two 500 mg doses of RTX evinced concentrations of up to 3.5 mg/L in the drained peritoneal dialysate (no details on the CAPD regimen were provided) (78). This case report, and the related theoretical calculations, suggests that when peritoneal dialysis is performed in patients treated with RTX, increased clearance should be considered and more frequent dosing may be required so as to maintain the treatment efficacy.

### Inflammation

A further factor that acts to influence the plasma protein levels and pharmacokinetics of MABs comprises inflammation, which may enhance the non-specific clearance of plasmatic proteins. This can be evaluated *via* the presence of low albumin levels of (non-nephrotic) inflammatory origin, which mirrors a high protein turnover and correlates negatively with the clearance of MABs (81).

## The variation in RTX pharmacokinetics in various glomerular nephropathies

The half-lives of RTX in various glomerular diseases and RA are listed in **Table 2** so as to provide a comparison of the pharmacokinetic alterations.

**Table 2:** The half-lives of RTX in various autoimmune diseases.

Drug/disease	Half-life
RTX/AAV	23 days = 552 hours (74)
RTX/MN	11.4 ± 5.4 days = 275 ± 130 hours (11) 11.5 days = 276 hours (82)
RTX/NS in children	23 days = 554 hours (34)
RTX/RA	19-22 days = 456-528 hours (62)

Only those studies that administered enough doses to saturate the target-mediated clearance (i.e. rather than single dose studies) were considered. AAV, ANCA associated vasculitis; MN, membranous nephropathy; NS, nephrotic syndrome; RA, rheumatoid arthritis.

### ANCA associated vasculitis

The average half-life of RTX in AAV patients is 23 days, which corresponds to the average half-life of this drug in most other diseases (74). Cornec et al. described highly variable pharmacokinetics in AAV patients, which are influenced by the gender and the new-diagnosis status. Males evinced 20% lower levels, which the authors ascribed to the increased volume of distribution (63). Nevertheless, other authors described also gender-dependent alterations in the RTX clearance that led to lower levels in males in a study with DLBCL patients (64). The Cornec et al. study revealed that patients with newly diagnosed AAV had lower RTX levels than patients with relapses, due probably to the higher “antigen burden” (the baseline levels of the circulating B-lymphocytes), which acted to increase the target-mediated clearance. Moreover, newly diagnosed patients evinced higher IgG levels and lower RTX levels following the 1^st^ RTX dose, which may prove the increased clearance of RTX due to the saturation of the FcRn receptors by high IgG levels. A slightly negative correlation was observed between RTX exposure and BSA even with BSA based dosing, whereas the BMI and age exerted no influence. Patients with lower RTX levels at week 2 with 4 times weekly 375 mg/m^2^ dosing exhibited a shorter time to B-cell repletion; however, this was not associated with worse clinical outcomes (63). This partially contrasts with another study that described the non-linear pharmacokinetics of RTX with a BMI dependent volume of distribution and a clearance that were influenced by the AAV disease type (associated with proteinase 3 (PR3) or myeloperoxidase (MPO) auto-antibodies) and the B-cell baseline count. The authors included target-mediated elimination and the “target latent antigen” (the number of CD20 molecules, as well as other unknown factors) in their pharmacokinetic model with the successful description of the RTX pharmacokinetics. The amount of latent antigen was four times higher in males and two times higher in newly diagnosed patients. The target-mediated elimination in AAV patients was three times lower than in NHL and seven times lower than in CLL. Generally, RTX led to a delayed decrease in ANCA antibodies, which were more sustained in MPO patients. The feedback exponent linked with the PR3 ANCA antibody was five times higher in patients with relapses, thus indicating that the levels of PR3-ANCA rebounded more rapidly in patients with relapsing disease (74).

### Membranous nephropathy

The variability in the efficacy of RTX in MN can largely be explained by the pharmacokinetic alterations to RTX in this disease. During MN treatment, the RTX half-life exhibits a negative correlation with urinary protein excretion and lasts a mere 11.5 days or so, which is shorter than in other autoimmune or hematologic diseases (11, 82). Fervenza et al. determined that compared to non-proteinuric RA patients, patients with MN-induced proteinuria had lower RTX levels. Nevertheless, the authors determined no differences between the RTX levels in the 8 responders and 7 non-responders in this small study (N=15) (83). The same authors proved that two 1000 mg doses administered 2 weeks apart and four weekly doses of 375 mg/m^2^ resulted in a similar drug exposure for MN patients, which is lower than in patients with RA without proteinuria. As early as on the 56^th^ day following the initiation of treatment, most of the MN patients evinced RTX levels of below the 5^th^ percentile of the levels measured in the RA patients. During the 2^nd^ treatment course 6 months later, the RTX levels were found to be significantly higher than during the first treatment cycle, which was most probably due to the reduced proteinuria. Nevertheless, no statistically significant correlation between the PU level and RTX levels was attained in this, admittedly, rather small study (n=20) (82). Chen et al. in their meta-analysis of studies that described the treatment of MN also proved that reduced proteinuria is associated with a better treatment response to RTX (84).

Boyer-Sauvet et al. in another study involving 43 MN patients, described the correlation between lower RTX levels 3 months following administration, poor B-cell depletion, high anti-phospholipase A2 receptor (anti-PLA2R) antibody titers and persistent proteinuria. A negative correlation between residual RTX levels at month 3 and anti-PLA2R antibody titers suggests the possible benefit of higher or repeated RTX doses in patients with the more active form of the disease. The authors suggested that the RTX level should be at least 1 mg/L at three months after administration so as to achieve long term remission following the first treatment course (85). This is in line with a study by Seitz-Polski et al. which compared two dosing regimens (2 x 1 g two weeks apart and 2 x 375 m^2^ one week apart) and determined a more rapid and effective response in patients that received the 2 x 1 g dose, who, moreover, also exhibited higher RTX levels at the third month of treatment, even though this may have been biased by the numerically (but not statistically significantly) higher level of PU in the comparison group with an approximately one fourth lower RTX dose (median PU 5.9 vs 8.4 g/day, respectively at enrolment). The PU correlated negatively with the residual RTX levels at the third month of treatment. The authors also proved the presence of lower levels of CD 19+ cells and anti-PLA2R antibodies at month 6 in the higher dose arm and proved that relapses correlated with incomplete anti-PLA2R depletion and so-called epitope spreading (i.e. when anti-PLA2R antibodies bind to more than one epitope on the target antigen) (86).

A further study examined the development of ADA in patients treated with RTX for MN. The authors observed the formation of ADA in 23% of treated patients and, even in those patients who initially responded to the treatment with the depletion of B-cells, the formation of ADA was linked to relapsing disease, increased PU and more rapid B-cell reconstitution. 8 of the 10 patients with ADA developed neutralizing ADAs and 2 of them were found to be cross reactive with other anti-CD 20 molecules. 3 patients with anti-RTX antibodies that did not bind ofatumumab were successfully treated with ofatumumab and 2 patients with non-neutralizing anti-RTX ADA were successfully treated with RTX (12).

### Minimal change disease/idiopathic nephrotic syndrome in children

There is a significant lack of pharmacokinetic data available on RTX in the treatment of MCD. According to a single dose study (375 mg/m^2^) on 12 children with steroid dependent NS, the half-life of RTX was 14.5 days. Most of the children developed B-cell depletion; however, relapses were frequent, with only 3 patients remaining relapse-free after 365 days. Proteinuria was not referred to (87). The data from another study involving 14 pediatric patients who received one or two RTX doses was used to compile a population pharmacokinetics model in which both the clearance and volume of distribution positively correlated with the BSA. The volume of distribution sustained from 2 compartments, one of which was BSA related and the other constant. The half-life calculated according to this model increased from 7.3 days in patients with a BSA = 1 m^2^ to 11.7 days in patients with a BSA = 2 m^2^. It is worthy of note that the patients had negative PU. Nevertheless, these values are still much lower than those reported for patients with RA and other non-nephrotic diseases (65). A further study, in which 48 Japanese children who were administered four weekly 375 mg/m^2^ doses following the remission of relapse induced by corticosteroids and various other immunosuppressants, reported an RTX half-life of 23 days (34). The differences in the PU do not explain the differences in the pharmacokinetic parameters between the former two and the latter study since in one of the studies that reported a remarkably short half-life the patients exhibited negative PU (65). Therefore, the longer half-life in the latter study may have been due to the patients having been given much higher doses, which may have acted to saturate the antigen-mediated clearance.

### Systemic lupus erythematosus

The pharmacokinetics of RTX have been studied to date in only one phase I/II dose escalating study involving 17 SLE patients (7 with LN). Very low mean RTX concentrations were determined 2 months following the administration of single doses of 100 mg/m^2^ and 375 mg/m^2^. 4 weekly 375 mg/m^2^ RTX doses led to a mean concentration of 9.4 mg/L after 2 months. There were several orders of magnitude between subject variations in the RTX levels in all the study arms and therefore, even though there were large differences between the doses, the RTX level ranges overlapped between the dosing groups (76).

### IgA nephropathy

RTX was abandoned early due to its infectivity in IgAN treatment (48); therefore, no studies have focused on the pharmacokinetic parameters of RTX with concern to this disease. Nevertheless, as the PU in more severe cases of IgAN becomes unselective (88), it should be noted that the degree of effectivity may be hampered by the loss of RTX in the urine for this patient group.

## Pharmacokinetic alterations and the response to treatment with RTX in glomerulopathies

The depletion of B-cells in response to RTX treatment has been observed to be very stable in AAV patients, and the pharmacokinetics are very similar to those of other autoimmune diseases that do not affect the kidneys (74). Nevertheless, profound pharmacokinetic changes occur in other glomerulopathies that may act to hamper the efficacy of RTX treatment.

The complete depletion of CD19+ cells during MN treatment is followed by their repopulation as soon as after 3 months. This is in line with the almost complete elimination of RTX after 3 months due to its short half-life in patients with non-selective nephrotic proteinuria. Nevertheless, Fervenza et al. were unable to prove that complete B-cell depletion is required for a clinical response in MN (82). A comparison of widely differing dosing protocols in a range of clinical studies revealed MN treatment responses of 44-87% at 24 months (33, 82, 83, 89). Even though the correlation between the RTX dose and the clinical response is difficult to determine *via* the comparison of different studies, Seitz-Polski et al. concluded that a higher dose (2 x 1 g) leads to more rapid remission; moreover, more patients evinced the remission of MN 6 months following treatment than did those who received lower doses (2x 375 mg/m^2^). No statistically significant differences were observed after 12 months, even though a numerically higher response rate for the group with higher RTX doses remained evident (86). It is important to note that the more rapid remission of nephrotic proteinuria is of significant clinical relevance since it limits the risk of nephrotic syndrome-related complications (including deep vein thrombosis). The need for higher RTX levels may be particularly important for those patients with MN with higher anti-PLA2R titers (90) and patients with anti-PLA2R that evince epitope spreading, which is linked to relapsing disease. The examination of epitope spreading is currently usually not performed; however, it has been shown that 95% of patients with anti-PLA2R titers of over 321 RU/mL are spreaders and, therefore, they may need to be treated with higher RTX doses (86). It has also been shown that with concern to patients with persistent anti-PLA2R positivity 6 months following initial RTX therapy, an additional RTX dose may act to overcome previous RTX unresponsiveness in MN treatment (91).

With respect to frequently relapsing and steroid dependent NS in children, which is similar to MCD in adults, the only available study that described RTX pharmacokinetics showed that following RTX-induced complete B-cell depletion, the B-cells started to repopulate after an average of 148 days. According to the mean RTX levels measured in this study, this corresponds roughly to a decrease in RTX serum levels to below 10 mg/L, the level required for induction of CDC. No relapse was registered during B-cell depletion for any of the patients in this study (34).

Since the overall relapse frequency is high during SLE maintenance treatment with RTX (92), new dosing approaches and the individualization of therapy are required. According to one small study, the depletion of B-cells during SLE treatment is more difficult to obtain for African Americans. Unfortunately, the authors did not study the pharmacokinetic differences between races and, therefore, it cannot be distinguished whether the poor response was due to the differing nature of the disease in African Americans or to the lower RTX exposure in these patients. Clinical improvement, as measured *via* the decrease in the SLAM score, was observed only for those with B-cell depletion and it was most notable in terms of the improvement of rashes, mucositis, alopecia and arthritis. Moreover, it was possible to decrease the doses of corticosteroids and other immunosuppressants for the patients involved. The overall stabilization of the kidney functions was observed for all the 7 patients with LN enrolled in the study, with one patient achieving complete remission (the disappearance of urinary casts and hematuria). Most interestingly, this study, which employed three different dosing schedules, revealed both good and poor responders regarding B-cell depletion in all the dosing groups, thus implying that the dose alone was not predictive of effectivity. Of note, the B-cell depleters evinced significantly higher RTX levels 2 months following administration regardless of the dose administered (76).

## Alternative non-RTX anti-CD20 therapies in glomerulopathies

A growing body of evidence suggests that for those patients who do not respond to RTX treatment, other anti-CD20 molecules may prove to be effective either due to their differing pharmacodynamic profiles or the bypassing of ADA that had been formed against RTX during previous treatment. Moreover, a small proportion of patients experience intolerance to RTX infusion despite the appropriate premedication (93); thus, alternative anti-CD20 molecules may provide a viable option for such patients. Of the various treatment modalities available, ofatumumab has been most studied to date in patients with immune-mediated nephropathies.

### ANCA associated vasculitis

Due to its excellent and reliable effectivity, over the last decade RTX has become the standard of care treatment option for induction therapy in patients with GPA or MPA, and is especially preferred in patients with severe or relapsing disease (24, 94). Nevertheless, the fully human anti-CD20 monoclonal antibody ofatumumab was administered along with low-dose cyclophosphamide and corticosteroids to eight patients with ANCA-associated vasculitis some of whom had been intolerant to previous RTX infusions. These patients successfully achieved disease remission with no unexpected side effects (95). In addition, an *in vitro* study using cells from patients with GPA indicated that obinutuzumab evinced a stronger effect than RTX with respect to both the reduction of B cells and the activation of NK cells due to the optimized Fc fragment-related activity of obinutuzumab (96). A RCT comparing the effectivity of obinutuzumab versus RTX for treatment of anti-PR3 positive ANCA associated vasculitis patients (NCT05376319) is ongoing.

### Membranous nephropathy

The discussion is ongoing as to the preference to treat MN patients with a high risk of progressive loss of kidney functions with alkylating cytostatics or RTX, with many researchers siding with the latter (97). However, approximately 30% to 40% of patients with MN do not respond to RTX (33). As shown above, the development of anti-RTX antibodies in patients with MN as a result of the chimeric nature of rituximab is closely associated with the reduced capability of RTX to induce and sustain disease remission. A study by Boyer-Suavet and others reported that three patients with anti-rituximab antibodies were successfully treated with the fully human anti-CD20 antibody ofatumumab (12). Moreover, a case report of a young male with MN sensitized against RTX also showed effective treatment with ofatumumab, which was both safe and cost effective (98). In another case series, patients with primary MN refractory to RTX were successfully treated with ofatumumab or obinutuzumab (99). The efficacy of obinutuzumab in the treatment of RTX-resistant primary MN has been described in three separate case reports (100). In addition, a pilot single-center study that reported on the use of obinutuzumab to treat patients with MN showed complete or partial remission in 6 of 7 (85.7%) RTX-refractory patients (101). Also ongoing phase II ORION study (NCT05050214) is testing the efficacy of obinutuzumab in primary MN. In rare cases, the coincidence of primary MN with multiple sclerosis has been known to occur, as in a recently published case of a 52-year-old patient. As demonstrated in this patient, the remission of both diseases can be achieved using ocrelizumab (102).

### Nephrotic syndrome in childhood

Increasing evidence of RTX resistance in children with NS has also stimulated research into alternative therapies (103). Concerning a case series of five pediatric patients with steroid-resistant NS resistant to all available standard drugs including RTX, remissions were achieved using ofatumumab (104). Another report concerning five pediatric patients with idiopathic NS showed that the application of ofatumumab resulted in complete remission in three patients and partial remission in one. However, one patient did not complete the ofatumumab treatment due to infusion reactions (105). A further study showed that low-dose ofatumumab infusion in childhood RTX-resistant NS induced remission in two patients with normal kidney functions, whereas it was unable to modify PU in another two patients with impaired kidney functions (106). The successful administration of ofatumumab was noted in two pediatric NS patients allergic to RTX (107). Fujinaga and Sakuraya also reported an effective single infusion of low-dose ofatumumab in one pediatric patient with complicated RTX-resistant NS with proven anti-RTX antibodies (108). However, a randomized placebo-controlled trial that compared low-dose ofatumumab and a placebo in multidrug-resistant pediatric NS was terminated for futility when all 13 randomized children remained nephrotic (109). Moreover, a recently published controlled randomized trial that compared ofatumumab to RTX in 140 patients with steroid-dependent and calcineurin inhibitor-dependent idiopathic NS showed the non-superiority of ofatumumab. The relapse rates in this study were comparable at 12 months (53% for ofatumumab and 51% for RTX) and non-significantly higher at 24 months (76% for ofatumumab and 66% for RTX) after a single dose infusion (110).

### Focal segmental glomerulosclerosis

A case series of seven patients who received ofatumumab for recurrent FSGS post-kidney transplantation demonstrated improvement in four out of five pediatric cases, with either complete or partial remission. However, ofatumumab administration led to no marked clinical improvement in the remaining two adult patients. No significant infusion reactions were noted in any of the patients (111). In a separate multicenter study, the treatment of the recurrence of NS following pediatric renal transplantation with ofatumumab led to partial remission in three out of six patients, with no response in the remaining three (112). Other small case reports of the post-transplantation recurrence of FSGS in children yielded positive results with the use of ofatumumab (113–115). Hopefully, more studies on other anti-CD20-depleting agents will be conducted in the same way as a recently registered study aimed at evaluating the safety and efficacy of obinutuzumab in adult primary FSGS (NCT04983888).

### Systemic lupus erythematosus

Whilst not officially approved by healthcare authorities for the treatment of SLE, RTX continues to be used in clinical practice for the treatment of refractory disease based on a considerable number of small open label studies and clinical experience publications that have presented the respective safety and efficacy data (116). However, two extensive randomized controlled trials concerning extra-renal lupus (the EXPLORER study) and LN (the LUNAR study) were unable to show the benefit of adding RTX on top of standard of care (44, 117). Therefore, other anti-CD20 depleting therapies were tested in patients with LN. Ocrelizumab was added on top of standard of care treatment in a large randomized controlled trial that evaluated the efficacy and safety of ocrelizumab in patients with class III/IV lupus nephritis (the BELONG study). Even though the overall renal response rates with ocrelizumab were numerically, but not statistically significantly, superior to those with a placebo, the study was terminated prematurely since ocrelizumab treatment was associated with a higher rate of serious infections (16). Interestingly, a phase II randomized controlled trial (NOBILITY) demonstrated improved renal responses with obinutuzumab in 102 patients with proliferative LN when it was added to the standard of care at 104 weeks of follow-up. Importantly, the incidence of serious infections or death did not increase with the administration of obinutuzumab (118). These results will be further evaluated in the phase III REGENCY trial (NCT04221477) and NCT02550652 trial that is comparing obinutuzumab + mycophenolate to placebo + mycophenolate in patients with LN. Furthermore, OBILUP study (NCT04702256) is comparing obinutuzumab + mycophenolate to corticosteroids + mycophenolate in order to test corticosteroid-free induction treatment of LN. Ofatumumab was also successfully used in case reports and series of patients for SLE patients who were intolerant to RTX (119–121). It was safely administered to 14 of 16 SLE patients with severe infusion reactions to RTX; however, 2 of these patients had infusion reactions also to ofatumumab and were unable to continue the treatment. After induction treatment all these patients were kept on maintenance therapy with mycophenolate. 12 patients in this cohort suffered from LN, 6 of whom showed complete renal remission after 6 months (121). A further two studies suggested that patients with SLE who did not respond to repeated RTX infusions for disease relapse should be treated with the alternative anti-CD20 antibodies ocrelizumab, obinutuzumab or ofatumumab (122, 123).

### Other glomerulopathies

Ofatumumab was administered as a potential corticosteroid-sparing agent to a patient with IgA vasculitis with nephritis with a previous allergic reaction to RTX, resulting in disease remission (124) even though RTX was found to be ineffective in a small randomized controlled study in patients with biopsy-proven immunoglobulin A nephropathy (48).

## The pharmacokinetics of other anti-CD 20 antibodies

No pharmacokinetic studies have been conducted for patients with glomerular disease treated with novel anti-CD20 therapy. Therefore, we describe here their pharmacokinetics in non-kidney affecting diseases based mainly on registration phase I, II and III studies including studies that described population pharmacokinetics. It is reasonable to expect that the variables that change the pharmacokinetics of these drugs are largely similar to RTX since the pharmacokinetics are less variable between different monoclonal antibodies than between chemical drugs.

The pharmacokinetics of ofatumumab have been described in four studies (2 with chronic lymphocytic leukemia, one with non-Hodgkin lymphoma and one with RA). The pharmacokinetic data from these studies was pooled so as to describe the population pharmacokinetics and the covariates that influence them. A population pharmacokinetic model was used to describe differences in the target-mediated clearance between diseases with markedly different B-cell numbers and turnover. The pharmacokinetics of ofatumumab was described *via* a 2 compartmental model with a Clearance = 7.5 mL/h; Volume of distribution = 5.5 L and T_1/2_ = 21.8 days. Target mediated clearance and the number of IgG (possibly due to the saturation of FcRn receptors) acted to increase the clearance and to shorten the T_1/2_. The other covariates that influenced the ofatumumab pharmacokinetics consisted of the BSA (the clearance and volume of distribution) and gender (a smaller volume of distribution in females) (125). Concerning chronic lymphocytic leukemia patients, a shorter half-life was measured during the first than subsequent infusions due probably to a decrease in the CD20 cell number. Higher exposure, a longer half-life and slower clearance all statistically significantly correlated with a better treatment response, which indicates that therapeutic drug monitoring may provide an approach to therapy individualization. On the other hand, this may merely reflect that a lower tumor burden that does not influence the pharmacokinetics of MAB may, to a large extent, predispose that patients will have a better response. The half-life was found to be longer with higher doses and after the fourth rather than the first infusion (126); a similar finding was also reported in a study that considered follicular lymphoma patients (20). The effect of target mediated clearance was less pronounced in RA (125). Concerning chronic lymphocytic leukemia patients in the highest dosing group (1x 500 mg followed by 3x 2000 mg weekly) the half-life was 13.6 days following the final infusion. The clearance was more rapid in males and in patients with a higher tumor burden (represented by the size of the lymph node measured as the sum of the product of the diameters). The volume of distribution was larger in males and correlated with the body weight and the BMI (126). In contrast, regrading follicular lymphoma patients, the half-life in the group with four weekly 1000 mg doses was 23.6 days and, therefore, more resembled that of RTX in non-nephrotic autoimmune diseases (20). Regarding follicular lymphoma, the treatment response failed to correlate with the anthropometric parameters, the ofatumumab dose, the pharmacokinetics or the overall exposition. Nevertheless, the exposition was found to be very variable for the patients in the treatment groups, and the AUC ranges overlapped even between the lowest and the highest dosing group, even though a 3 times higher dose was applied in the latter. Only one patient developed transient ADA against ofatumumab in this follicular lymphoma trial (20).

A robust population pharmacokinetics study with 4901 samples from 941 subjects was performed with ocrelizumab in patients with multiple sclerosis. The B-cell depletion was observed to be more frequent in the higher exposure quartiles. The clearance and volume of distribution increased with the body weight; the clearance was also more rapid in patients with higher B-cell numbers, and the volume of distribution was larger in males. The overall exposure was lower for patients > 90 kg and higher for patients < 60 kg compared to the 60-90 kg group. The half-life was 26 days for average 75 kg women (127).

The pharmacokinetics of veltuzumab were described in a phase I/II study in patients with hematological diseases. The half-life was determined at between 13.3 and 19.7 days, without more details on the pharmacokinetics being provided. The overall exposure of veltuzumab in responders with follicular lymphoma was higher than in non-responders (128).

The population pharmacokinetics for obinutuzumab with respect to hematological diseases were derived from data pooled from 6 studies involving a total of 961 patients. The population pharmacokinetics model comprised 2 compartments (central = 2.72 L and peripheral = 1.23 L). The clearance consisted of the time independent component and the time dependent component, which progressively decreased due to a decrease in the target mediated clearance. As in other anti-CD20 molecules, the target mediated clearance apparently plays a different role in different diseases. This was reflected in the differing rate of the decline in the clearance, which was most rapid in follicular lymphoma and slowest in marginal zone lymphoma. The initial value of the time dependent clearance component was higher in patients with larger tumor sizes. The overall exposure correlated positively with the efficacy and no concentration dependent safety issues were observed (68). Whether the obinutuzumab pharmacokinetics in autoimmune diseases are similar to those in certain hematological diseases and whether pharmacokinetic issues play a role in the optimization of treatment schemes remain to be determined. A study on the pharmacokinetics of obinutuzumab pharmacokinetics in SLE nephritis is currently underway (NCT05039619).

## Discussion

The pharmacokinetics of RTX and other anti-CD20 molecules differ markedly between hematologic and autoimmune diseases due especially to alterations in the B-cell numbers and the target-mediated clearance component (58, 63, 125). The clearance of RTX may also be altered in nephrology patients due to increased losses *via* the urine in the non-selective PU period (77), elimination *via* therapeutic plasma exchange (59, 80) or, possibly, through peritoneal dialysis (78). RTX evinces a reliable degree of efficacy and pharmacokinetics that are similar to other autoimmune diseases in AAV, where it represents an effective first-line induction treatment (27–29). To date, not enough evidence is available to suggest a preference for newer anti-CD20 monoclonal antibodies in patients with GPA or MPA over RTX, although they most probably represent a safe alternative for patients who are intolerant to RTX. RTX has also been shown to be effective in MCD (35, 36). In contrast, RTX treatment for IgA GN was found to be ineffective; moreover, this was probably not caused by the elimination of the drug in the urine since patients experienced sustained 12 month-long depletion (48). Even though RTX also appears to be effective in MN and LN, its role is ambiguous at best due to relatively low response rates, which may be caused by the pharmacokinetics or pharmacodynamic aspects (26, 44).

RTX pharmacokinetics are markedly altered in MN with an increase in the clearance due to the loss of RTX in the urine (77). All the published MN dosing instructions share the same recommendation to administer one-to-four doses over a short time period and to wait several months before administering another dose (33, 82, 83, 89). According to the findings of Fervenza et al., who showed that neither peak nor trough RTX levels correlated with the response to RTX therapy in MN patients (82), it appears that neither the monitoring of drug levels nor other dosing individualization approaches are feasible. On the other hand, the data could also be interpreted as MN patients with relatively short RTX half-lives may not need so high loading doses; however, at least at the beginning of treatment, it appears that they might benefit from more frequent (e.g. monthly) dosing with lower maintenance doses. This regimen would act to prevent RTX levels from declining to below the critical value during the dosing interval. While the exact critical value remains to be established, according to hematological studies it is likely to be 10 mg/L in serum and 25 mg/L in whole blood. These levels are required for RTX to trigger CDC, with much lower levels needed to trigger other RTX modes of action (58). Following an initial “dense dosing period” the dosing interval may be prolonged if the PU decrease as nephrotic PU is thought to be the reason for the shortened RTX half-life in MN (11, 82). If this is the case, the appropriate dosing individualization can be determined *via* therapeutic drug monitoring methods. Before this approach is confirmed as advantageous, a potential treatment efficacy marker for dosing frequency adjustments comprises the anti-PLA2R levels in patients who are anti-PLA2R positive since it seems that an increase in anti-PLA2R antibody levels provides the most sensitive marker for the failure of RTX treatment. Current KDIGO guidelines recommend to administer another RTX dose in patients that are anti-PLA2R positive 6 months following the initiation of treatment (24). The more frequent dosing of smaller doses may also be beneficial due to the prevention of trogocytosis (“CD20 shaving”) during the period of high RTX levels following the administration of large doses (58, 72). With regard to alternative anti-CD20 therapy, it seems that it is effective in patients with primary MN refractory to RTX or RTX intolerance and, therefore, may represent a treatment option for such patients. However, there remains a lack of data from randomized controlled trials that would serve to confirm this hypothesis. An ongoing trial (NCT04629248) is currently comparing obinutuzumab with tacrolimus in patients with MN.

The pharmacokinetic variability together with the fact that the B-cell depletion correlated with a higher RTX exposition suggests that therapeutic drug monitoring provides a meaningful approach to the tailoring of treatment for SLE patients. Nevertheless, there are a number of important issues that still need to be addressed regarding the pharmacodynamics of RTX in SLE patients. A small SLE study proved that patients heterozygous with low affinity FcγIIIR alleles needed higher RTX levels so as to obtain B-cell depletion (15). Moreover, it has been shown that the complement may be depleted if RTX is administered frequently during the treatment of hematological malignancies and that this may subsequently result in the diminished efficacy of RTX if CDC cannot be triggered (58). Complement elements may be depleted in the SLE due to pathologic complement activation either due to the disease itself or the formation of anti-complement antibodies (129). Since CDC has been described as being crucial with respect to RTX effectivity (20, 21), the low effectiveness in LN patients may be caused by their low complement molecule levels. CDC is increasingly being recognized as the crucial mechanism of RTX and of certain other anti-CD20 antibodies that may be more effective than RTX especially in patients with low CD20 expressing diseases. Since RTX dissociates from CD20 relatively rapidly, it may fail to bind the C1q component of the complement and activate the complement cascade (20, 21). In the case of significant SLE-induced hypocomplementemia, this may be even more pronounced and could result in the partial loss of efficacy, a hypothesis that still remains to be proven. In addition, the effector cells needed for ADCC may be exhausted following high exposure to RTX with initial frequent dosing (58). It is probable that the effectivity of the treatment of LN can be improved by switching to another anti-CD20 molecule. In this regard, ofatumumab may be the best choice due to its longer residence time, which allows for the firmer binding of C1q (20), nevertheless, obinutuzumab currently appears to perform best in terms of the clinical response of the various alternative anti-CD20 antibodies (118, 130).

Since the effectivity and B-cell depletion of RTX and other anti-CD20 molecules has repeatedly been shown to correlate with the plasmatic levels of the drugs in different diseases (68, 76, 126, 128), therapeutic drug monitoring may offer a significant advantage when tailoring therapy for individual patients. Nevertheless, the pharmacokinetic treatment targets still need to be determined in patients with glomerulopathies.

## Author contributions

JH and VK performed literature review and prepared the manuscript. ZH and VT reviewed the article and provided suggestions and corrections regarding the field of nephrology. OS reviewed the article and provided suggestions and corrections regarding the field of pharmacology. All authors contributed to the article and approved the submitted version.

## Funding

This study was supported by research initiatives of the Ministry of Health of Czech Republic RVO-VFN 64165 and Ministry of Education, Czech Republic grant COOPERATIO 207034 Internal Disciplines and Pharmaceutical sciences.

## Conflict of interest

The authors declare that the research was conducted in the absence of any commercial or financial relationships that could be construed as a potential conflict of interest.

## Publisher’s note

All claims expressed in this article are solely those of the authors and do not necessarily represent those of their affiliated organizations, or those of the publisher, the editors and the reviewers. Any product that may be evaluated in this article, or claim that may be made by its manufacturer, is not guaranteed or endorsed by the publisher.
